# A novel single amino acid deletion impairs fibronectin function and causes familial glomerulopathy with fibronectin deposits: case report of a family

**DOI:** 10.1186/s12882-019-1507-7

**Published:** 2019-08-16

**Authors:** Maria Luíza Gonçalves dos Reis Monteiro, Fabiano Bichuette Custódio, Precil Diego Miranda de Menezes Neves, Frederico Moraes Ferreira, Elieser Hitoshi Watanabe, Antônio Marcondes Lerário, Liliane Silvano de Araújo, Bruno Eduardo Pedroso Balbo, Vívian Christine Dourado Pinto, Lívia Maria Gruli Barbosa, Vilmar de Paiva Marques, Juliana Reis Machado, Marlene Antônia Reis, Luiz Fernando Onuchic

**Affiliations:** 1 Nephropathology Service, Federal University of Triângulo Mineiro, Praça Manoel Terra, 330 – Bairro Abadia, Uberaba, MG 38015-050 Brazil; 2 Nephrology Service, Federal University of Triângulo Mineiro, Uberaba, MG Brazil; 3 Divisions of Nephrology and Molecular Medicine, University of São Paulo School of Medicine, São Paulo, SP Brazil; 4 Laboratory of Immunology, Heart Institute, University of São Paulo, São Paulo, SP Brazil; 5 Division of Endocrinology, University of Michigan, Ann Arbor, USA

**Keywords:** Fibronectin deposits, Glomerulopathy, Pathogenic mutation, Protein structure analysis; whole exome sequencing

## Abstract

**Background:**

Glomerulopathy with fibronectin deposits is an autosomal dominant disease associated with proteinuria, hematuria, hypertension and renal function decline. Forty percent of the cases are caused by mutations in *FN1*, the gene that encodes fibronectin.

**Case presentation:**

This report describes two cases of Glomerulopathy with fibronectin deposits, involving a 47-year-old father and a 14-year-old son. The renal biopsies showed glomeruli with endocapillary hypercellularity and large amounts of mesangial and subendothelial eosinophilic deposits. Immunohistochemistry for fibronectin was markedly positive. Whole exome sequencing identified a novel *FN1* mutation that leads to an amino-acid deletion in both patients (Ile1988del), a variant that required primary amino-acid sequence analysis for assessment of pathogenicity. Our primary sequence analyses revealed that Ile1988 is very highly conserved among relative sequences and is positioned in a C-terminal FN3 domain containing heparin- and fibulin-1-binding sites. This mutation was predicted as deleterious and molecular mechanics simulations support that it can change the tertiary structure and affect the complex folding and its molecular functionality.

**Conclusion:**

The current report not only documents the occurrence of two GFND cases in an affected family and deeply characterizes its anatomopathological features but also identifies a novel pathogenic mutation in *FN1*, analyzes its structural and functional implications, and supports its pathogenicity.

## Background

Glomerulopathy with fibronectin deposits (GFND) is a rare autosomal dominant heterogeneous disorder, characterized by proteinuria, hematuria, hypertension and, potentially, progression to renal failure [1]. This disease is caused by pathogenic mutations in the fibronectin-encoding *FN1* gene, located in chromosomic region 2q34 - when the disorder is named GFND2 [2, 3] - or in a locus mapped to 1q.32 (GFND1) [3]. FN1 is a 2386-amino-acid (aa) protein with a combination of FN1 type I, II and III domains (FN1, FN2 and FN3, respectively), involved in cell adhesion, motility, shape maintenance, opsonization, and wound healing. GFND manifests more often in the third or fourth decade of life [3], although this may occur at other age ranges. Most patients have normal renal function at diagnosis, usually advancing to end-stage kidney disease (ESKD) 15–20 years after clinical onset [4].

In this report, we present two cases of GFND, father and son, with nephrotic syndrome, hematuria and hypertension associated with a novel *FN1* mutation. This variant consists of an in-frame deletion that most likely leads to a single aa loss, a variant that required primary amino-acid sequence analysis to assess its potential pathogenicity. This evaluation supported its deleterious effect.

## Case presentation

A 14-year-old male was admitted with blood pressure of 165/100 mmHg, generalized edema, ascites, serum albumin of 1.4 g/dL, 24-h proteinuria of 5.42 g, hematuria 1+/4+ and serum creatinine of 0.73 mg/dL, reflecting an estimated glomerular filtration rate (eGFR) of 138 mL/min/1.73m^2^ using the Chronic Kidney Disease Epidemiology Collaboration (CKD-EPI) formula. Antineutrophil cytoplasmic, anti-nuclear and anti-DNA antibodies were negative, as well as serologies for hepatitis B and C, syphilis and human immunodeficiency virus (HIV). C3 and C4 levels were normal.

**Fig. 1:**
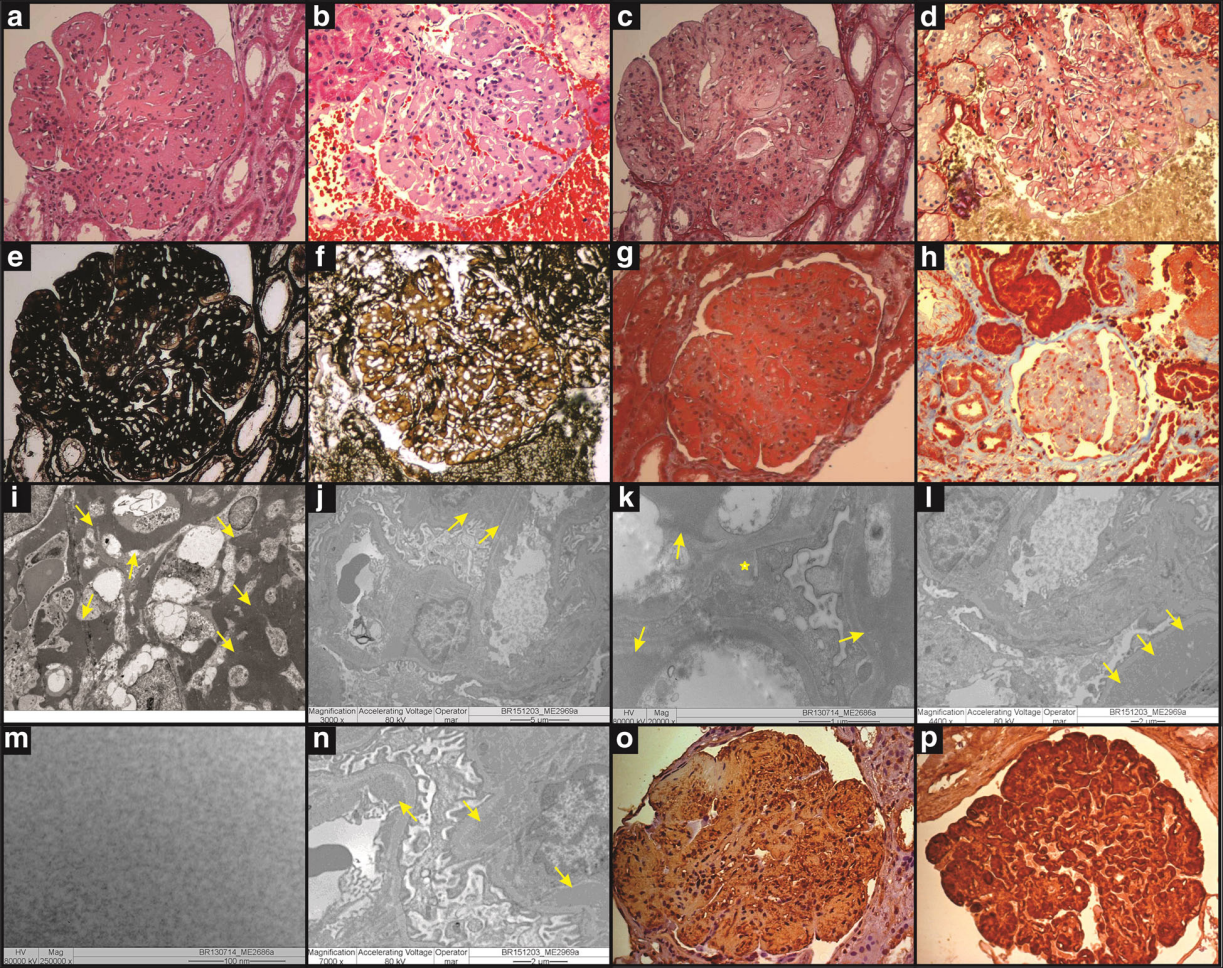
Renal histological and ultrastructural findings in the child (**a**, **c**, **e**, **g**, **i**, **k**, **m** and **o**) and his father (**b**, **d**, **f**, **h**, **j**, **l**, **n**, **p**), affected by Glomerulopathy with Fibronectin Deposits. **a** and **b** - Glomeruli with increased size, lobed, with endocapillary hypercellularity mainly due to mesangial cells. Eosinophilic, homogeneous coarse deposits in mesangial and subendothelial regions (HE, 20x obj.). **c** and **d** - The referred deposits are negative to Sirius red staining (SR, 20x obj.). **e** and **f** - Deposits are also negative to silver impregnation while areas with double contour of capillary loops can be observed (PAMS, 20x obj.). **g** and **h** - The deposits, on the other hand, are positive to Masson’s trichrome stain (TRI, 20x obj.). **i** and **j** - Electron microscopy shows massive electron-dense deposits in mesangium (**i**) and subendothelial location (**j**), identified by arrows (original magnification, × 3000). **k** and **l** images show abundant electron-dense deposits in mesangium and capillary loops, predominantly subendothelial but focally subepithelial in K (identified with a star) (original magnification, × 20,000 in **k** and × 4400 in **l**). **m** and **n** - In high-power view, deposits are finely granular (**m** - original magnification, × 250,000) and in subendothelial location (**n** - original magnification, × 7000). **o** and **p** - Immunohistochemistry for fibronectin revealing markedly positive giant deposits in mesangium and capillary loops (IH for fibronectin, 20x obj.)

Renal biopsy showed all glomeruli with mesangial hypercellularity and large amounts of eosinophilic fuccinophilic deposits, while are negative for methenamine silver and Sirus red in mesangium and subendothelium (Fig. 1A-H). Some capillary loops had basement membrane double contour. Absence of Congo red staining excluded amyloidosis, and immunofluorescence for IgA, IgG, IgM, Kappa, Lambda Transmission-electron microscopy (TEM), C1q and fibrinogen were negative. Analysis revealed massive electron-dense deposits, granular and globally present in mesangium, occasionally subendothelial and rarely subepithelial. There were foci of mesangial interposition, duplication of basement membrane and foot process effacement (Fig. 1I-N). Immunohistochemistry showed marked fibronectin staining in glomerular deposits, establishing the diagnosis of GFND (Fig. 1O-P). No consanguinity was identified and treatment was initiated with angiotensin I-converting enzyme inhibitor (ACEi). Renal function remained stable but daily proteinuria persisted between 3.0–5.0 g after three years.

To fully characterize his medical condition, whole exome sequencing was carried out and detected the novel c.5962_5964del:p.1988_1988del variant in *FN1*. This variant was confirmed by Sanger sequencing. Mutation Taster and SIFT softwares were used to assess its potential pathogenicity, however were not robust enough to yield clear results given the in-frame deletion pattern. This variant, however, was not found in the ExAC, 1000G and our local databases, and the deleted aa is conserved in mammalian and several non-mammalian species. In addition to the Ile1988 deletion, such programs also brought a less likely possibility of splice change, with uncertainty about potential protein sequence changes/truncations. Since the Ile1988 deletion is present in both situations, and the last possibility includes additional changes to the protein, pathogenicity analysis directed to p.1988_1988del was the essential evaluation to be performed.

**Fig. 2:**
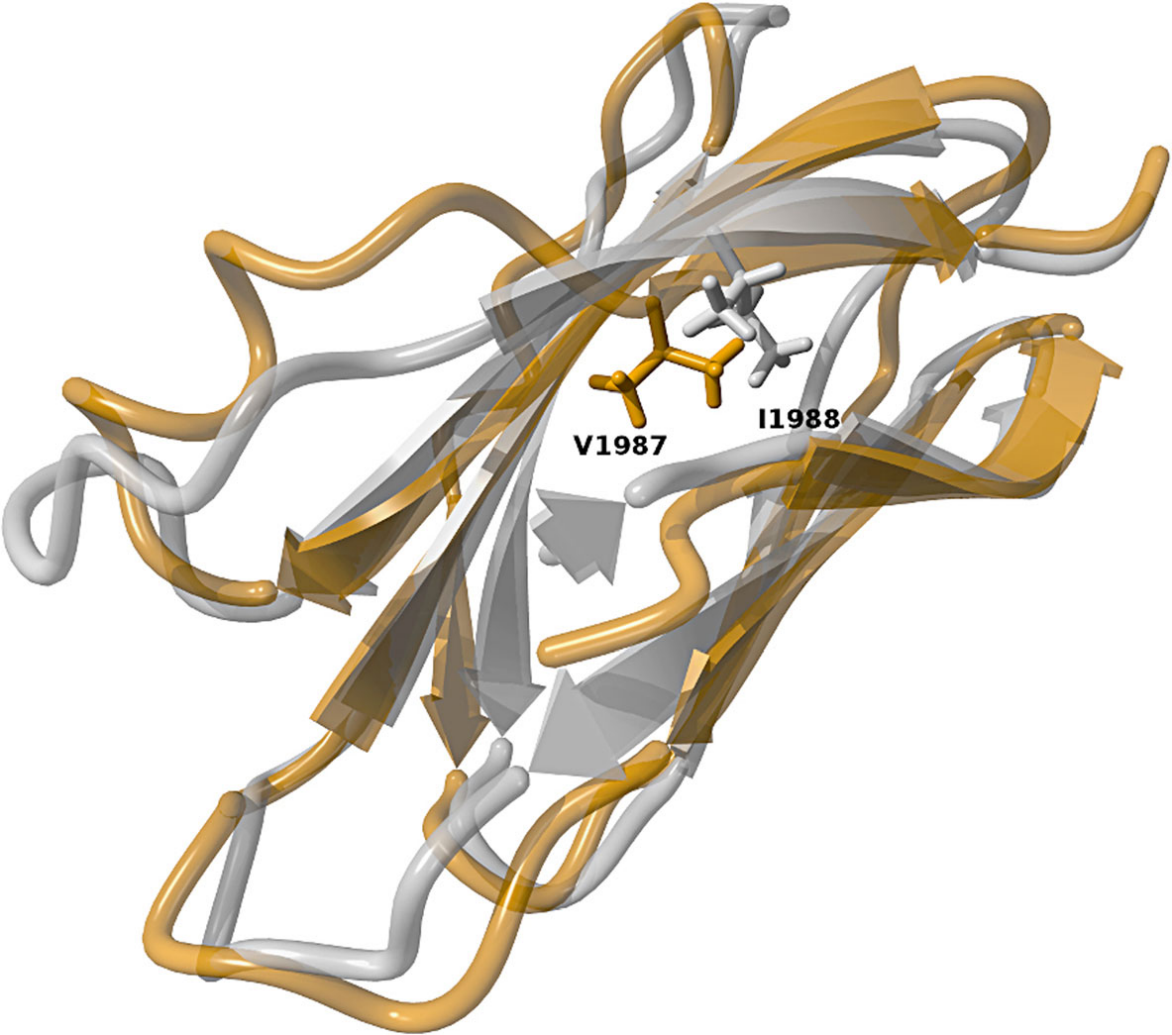
Superimposition between the native (white) and mutated (bronze) FN3 domain models. Despite the replacement of the native Ile1988 position with Val1987 in the mutated model, the previous eight residues had their main chain trace completely altered, as well as in other parts of the molecule. Several contacts were lost so that the mutated model is floppier

Given the limitations of the prediction softwares based on DNA sequence, we performed primary amino-acid sequence analysis directed to the Ile1988 deletion in three levels of complexity to assess the variant likely deleterious effect. The first one was based on sequence alignment. Ile1988 deletion is positioned in the 15th fibronectin type III (FN3) domain at the C-terminus. 1273 FN1 relative sequences available in the National Center for Biotechnology Information (NCBI) Reference Sequence database were clustered into 66 representative sequences and aligned [5]. The resulting alignment showed a hydrophobic residue at position 1988 in far most sequences, revealing that the Ile1988 residue is highly conserved among all FN1 sequences. At the second level, the pathogenicity of this deletion was evaluated with PROVEAN [6], yielding a deleterious effect score of − 7.053. Score values below − 2.5 are consistent with prediction of a deleterious effect. The third level and more complex analysis included tertiary FN3 structural analysis using molecular mechanics. This evaluation included wild-type and mutated models, modeled with Yet Another Scientific Artificial Reality Application (YASARA) [7] suite using the human FN3 crystal structure as template (PDB 3R8Q) and submitted to simulations in explicit solvent. 16,000 trajectories of 100 ps were calculated with the Yet Another Model Building and Energy Refinement force field 3 (YAMBER3 force field) and recorded for each wild-type and mutant molecule. Comparison analysis revealed that absence of Ile1988 leads to replacement with Val1987 at the molecular level. As consequence, the upstream Leu1977-Val1986 residues had their main chain trace completely altered, losing their beta-strand conformation present in the original molecule. In the native FN3 domain, the β-sheet conformation, composed of four anti-parallel β-strands, is maintained by a hydrogen bond network and hydrophobic interactions which are partially disrupted in the absence of Ile1988. The simulation shows, in fact, that in the mutated FN3 domain several hydrogen bonds between the strand main-chain nitrogens and the adjacent strand main-chain oxygens are lost or assumed a non-optimal distance, disrupting a series of hydrophobic contacts, increasing the flexibility of the whole domain and jeopardizing the domain structural stability (Fig. 2). These results are consistent with a floppy mutated subunit, which may affect the folding of the complex and result in a defective molecule. Altogether, these results strongly support that the functional activity of the mutated FN1 might be impaired or lost.

**Fig. 3:**
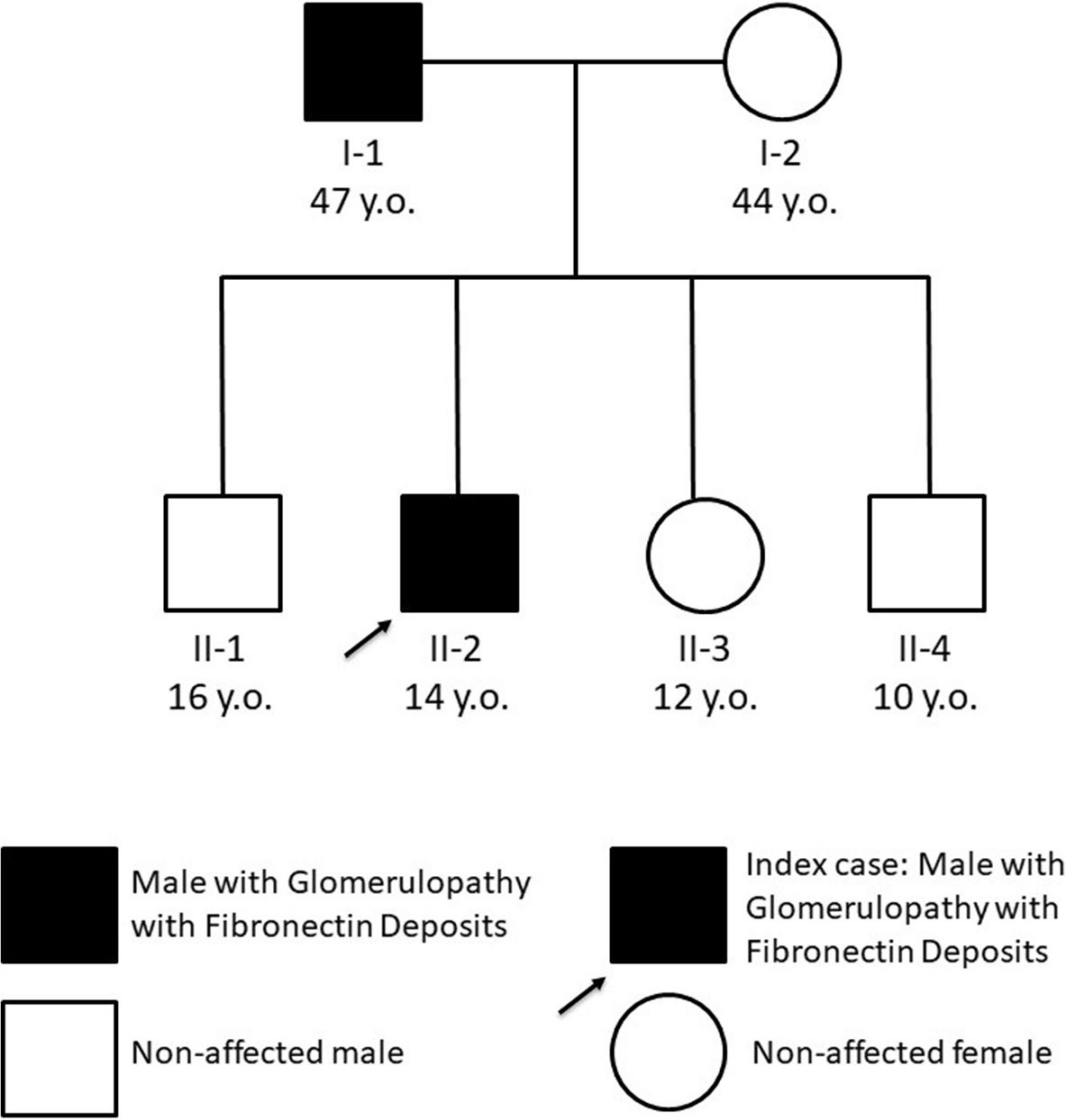
GFND family pedigree

The patient’s father was found to have proteinuria of 4.98 g/day, hematuria and hypertension at 47 years of age, being placed on the angiotensin receptor blocker (ARB) losartan. Search for secondary causes of nephrotic syndrome was negative. After losing his nephrology follow-up and two years after his son diagnosis, proteinuria was 5.0 g/day and serum creatinine 1.2 mg/dL (eGFR of 72 mL/min/1.73m^2^). Renal biopsy showed similar findings to his son’s. Marked fibronectin staining coincided with mesangial and capillary loop deposits, especially in subendothelial space, also establishing the diagnosis. He is currently on a higher dose of losartan and remains with similar levels of proteinuria. As expected, the c.5962_5964del:p.1988_1988del variant was also found in the father but not detected in the non-affected mother, two brothers and sister (Fig. 3). No proteinuria or renal dysfunction was detected in the patient’s mother and three siblings.

## Discussion and conclusions

GFND was originally described as a familial glomerulopathy with fibrillar giant deposits [8]. Later demonstration of fibronectin immunoreactivity led to the term famillial glomerulonephritis with fibronectin deposits [3]. More recently, the disease was associated with mutations in *FN1*, accounting for about 40% of the cases. As observed in our patients, typical findings include eosinophilic, homogeneous and coarse deposits in mesangial and subendothelial regions, negative to Congo red, silver and Sirius red, and positive for Masson’s trichrome and periodic acid Schiff (PAS) staining. Glomeruli are increased in size with lobed appearance, have endocapillary hypercellularity mainly due to mesangial cells and some areas exhibit capillary loop double contour. Immune complex deposition is classically absent [1]. IgM and C1q in mesangium has been reported, however such findings have been assigned to insulation of these proteins. Electron-dense deposits in mesangial and subendothelial regions are typically seen, most often finely granular and rarely fibrillary [1]. When present, fibrils are 9–16 nm in diameter and randomly distributed [9].

Diagnostic confirmation requires, in addition to the TEM findings, strong plasma fibronectin isoform staining in mesangium and capillary loops. Diagnosis of GFND may be challenging, however, particularly in the absence of clinical suspicion, family history and previous serum tests. Accurate molecular diagnosis, in turn, is currently available for GFND2 [9].

Fibronectin is an extracellular matrix glycoprotein with various homologous aa sequences and distinct motifs with specific domains to bind heparin, collagen, fibrin and DNA [10]. It includes two 250-kDa units with type I, II and III domains held together by disulfide bridges. This molecular structure allows it essential roles in several biological processes, including interaction between cells and extracellular matrix [11]. Fibronectin has two isoforms; one cellular, insoluble, and another circulating, soluble [1]. The cellular form is produced by glomerular mesangial cells and, in other tissues, mainly by fibroblasts [10]. The circulating form is synthesized by hepatocytes and can deposit in glomeruli [3, 12]. Our primary sequence analyses showed that Ile1988 is very highly conserved among relative sequences and is positioned in a key FN3 domain, a 90-residue segment in the protein C-terminus containing heparin-binding and fibulin-1-binding domains [13]. Heparin is a mucopolysaccharide sulfuric acid ester with anticoagulation properties while fibulin-1 is a glycoprotein found in connective tissues, some basement membranes and elastic fibers [14]. The c.5962_5964del:p.1988_1988del mutation was predicted to be deleterious and performed simulations suggest that it can change the molecular structure of the whole domain and possibly affect the folding of the complex. Such observations strongly support pathogenicity for this mutation.

In line with our results, the previously described *FN1* variants W1925R and L1974R, which affect the same HepII region, were shown to have less affinity to Heparin and were associated with impaired capacity of inducing endothelial cell spreading and cytoskeleton reorganization [2]. In addition, fibulin, the other protein that interacts with the region where Ile1988del lies, has been associated with chronic kidney disease [14].

The mechanism of fibronectin glomerular deposition is poorly understood [15]. The most likely hypothesis is a defective clearance of metalloproteinases and abnormal conformation of plasma fibronectin [3], which disseminates into tissues and incorporates into the fibrillar matrix [3, 12]. By modifying fibril formation, mutations in *FN1* can also lead to isoform imbalance, allowing plasma fibronectin incorporation into the glomerular nonfibrillar matrix [2]. Moreover, fibronectin produced in mesangium could also partially contribute to the deposits [16].

Amyloidosis is the most common glomerulopathy with fibrillary deposits. Differential diagnoses also include cryoglobulinemia, fibrillary, immunotactoid and collagenofibrotic glomerulonephritis. Distinction is primarily based on the deposit ultrastructural features, if filamentous or with microtubular formation [15], and on the fibril diameter: 8–12 nm in amyloidosis; 18–22 nm in fibrillary glomerulonephritis, 10–90 nm in immunotactoid glomerulonephritis; and 43–65 nm in collagenofibrotic glomerulonephritis [4]. Given the paucity of filamentous structures, the diagnosis of GFND can be occasionally made by immunoelectron microscopy [15].

There is no specific treatment for GFND. However, if diagnosed early, improvement in clinical status may be achieved at least temporarily with drugs, especially ACEis or ARBs [1]. Such agents were used in our cases, being associated with relatively preserved renal function but maintained nephrotic-range proteinuria. Although the prognosis of GFND is unknown [15], ESKD cases can be submitted to any renal replacement therapy, including transplantation [1]. Recurrence is possible, however, given the persistence of abnormal circulating fibronectin [4], but additional studies are needed to determine this risk.

The current report not only documents the occurrence of two GFND cases in an affected family and deeply characterizes its anatomopathological features but also identifies a novel pathogenic mutation in *FN1*, analyzes its structural and functional implications, and supports its pathogenicity.

## Data Availability

All meaningful data generated or analyzed in this study are included in the manuscript.

## Abbreviations

ACEi: Angiotensin I-converting enzyme inhibitor
ARB: Angiotensin receptor blocker
CKD-EPI: Chronic Kidney Disease Epidemiology Collaboration
eGFR: Estimated glomerular filtration rate
ESKD: End-stage kidney disease
FN3: Fibronectin type III
GFND: Glomerulopathy with fibronectin deposits
HIV: Human immunodeficiency virus
NCBI: National Center for Biotechnology Information
PAS: Periodic acid Schiff
PROVEAN: Protein Variation Effect Analyzer
TEM: Transmission-electron microscopy
YAMBER3 force field: Yet Another Model Building and Energy Refinement force field 3
YASARA: Yet Another Scientific Artificial Reality Application
